# Hybridized plasmon modes and near-field enhancement of metallic nanoparticle-dimer on a mirror

**DOI:** 10.1038/srep30011

**Published:** 2016-07-15

**Authors:** Yu Huang, Lingwei Ma, Mengjing Hou, Jianghao Li, Zheng Xie, Zhengjun Zhang

**Affiliations:** 1 State Key Laboratory of New Ceramics and Fine Processing, School of Materials Science and Engineering, Tsinghua University, Beijing 100084, P. R. China; 2 High-Tech Institute of Xi’an, Shaanxi 710025, P. R. China; 3 Key Laboratory of Advanced Materials (MOE), School of Materials Science and Engineering, Tsinghua University, Beijing 100084, P. R. China

## Abstract

For the attractive plasmonic structure consisting of metal nanoparticles (NPs) on a mirror, the coexistence of near-field NP-NP and NP-mirror couplings is numerically studied at normal incidence. By mapping their 3D surface charge distributions directly, we have demonstrated two different kinds of mirror-induced bonding dipole plasmon modes and confirmed the bonding hybridizations of the mirror and the NP-dimer which may offer a much stronger near-field enhancement than that of the isolated NP dimers over a broad wavelength range. Further, it is revealed that the huge near-field enhancement of these two modes exhibit different dependence on the NP-NP and NP-mirror hot spots, while both of their near-field resonance wavelengths can be tuned to the blue exponentially by increasing the NP-NP gaps or the NP-mirror separation. Our results here benifit significantly the fundamental understanding and practical applications of metallic NPs on a mirror in plasmonics.

Near-field enhancement is one of the most remarkable phenomena associated with nobel metals in the visible region. When light interacts with metals, collective oscillations of the conduction electrons can be excited at the metal surface, which are known as surface plasmons (SPs)^1^. As a result, local electric fields within the metal nanostructures can achieve strengths which are orders of magnitude higher than that of the incident field. This unique feature serves as the fundamental mechanism for a wide variety of applications such as surface enhanced spectroscopies^2^,^3^,^4^,^5^,^6^, chemical and biological sensing^7^,^8^,^9^, single molecule detection^10^,^11^, nonlinear optics^12^,^13^,^14^, to name a few.

One of the most geometrically simple but plasmonically important structures is the dimer, consisting of two metal nanoparticles (NPs) separated by a nanoscale gap^15^,^16^. Usually, dipole plasmons of individual NPs hybridize to form the bonding dipole plasmon (BDP) mode at lower energies, giving rise to enormous electromagnetic (EM) field enhancement at the nanogap, i.e., the “hot-spot”^17^,^18^. Local EM fields can be further enhanced by narrowing the gaps until reaching the quantum tunneling region^19^. Yet, despite the continuous progress of nanofabrication techniques, the production of dimers with reproducible and controllable nanogaps remains a challenge nowadays, especially for gap dimensions under 10 nm^20^,^21^.

Alternatively, one closely related system composed of metal NPs positioned over a thin metal film has recently received an increasing amount of attention^22^,^23^,^24^,^25^. In the nanoparticle-on-mirror (NPOM) structures^26^,^27^,^28^, the NP couples with its mirror image in the metal film, which can be understood as the hybridization between the localized surface plasmon of the NP and the propagating surface plasmon polariton of the metal surface^29^,^30^,^31^. Within the NP-mirror gap region, the EM fields is strongly enhanced, generating a hot-spot. In addition, the NPOM structures can be easily fabricated over large areas using well-developed top-down foundry processes instead of expensive and time-consuming e-beam based nanofabrication. The NP-mirror separation distance can be well-tuned by adjusting the thickness of the dielectric spacer down to nanometer and even subnanometer scale, resulting in both the tunable resonance positions and highly uniform, reproducible hot-spots^32^,^33^,^34^,^35^,^36^. All these advantages make the NPOM system a promising platform for surface enhanced Raman scattering (SERS)^27^,^28^,^33^,^34^, plasmon enhanced photoluminescence^37^,^38^, surface enhanced fluorescence^39^, plasmon-driven surface catalysis^40^, and related electronic effects.

So far, beside the thickness of the dielectric spacer, the NPOM system is also found to be remarkly sensitive to various other factors, including the angle of incidence, the surrounding medium, the NP size and material^27^,^39^,^41^,^42^,^43^,^44^. However most of these studies are focused on the dipole response of single NP on the mirror. For multi-particle configurations above the mirror including the NPOM structure and the structure for shell-isolated nanoparticle-enhanced Raman spectroscopy (SHINERS)^2^,^27^,^28^, both of the NP-NP and NP-mirror couplings contribute to the total near-field enhancement. A lot of effort has been put into the precise control over the locations of hot spots and the brilliant spectroscopy applications^45^,^46^,^47^,^48^,^49^,^50^,^51^,^52^,^53^. Understanding and predicting the plasmon hybridizations present in these complicated system are both necessary to realize and fully optimize potential plasmonic devices.

In this paper, the coexistence of near-field NP-NP and NP-mirror couplings at normal incidence is numerically considered using a NP-dimer on a mirror (NPDOM) model. The plasmon hybridizations are investigated by varying the gap width of the NP dimer and the thickness of the dielectric spacer. Although the incident angle can be optimized to achieve a maximum near-field enhancement^27^,^39^, the normal illumination is widely used in practical applications, especially for portable Raman spectrometer^2^,^24^,^28^. To be specific, we demonstrate two different kinds of BDP modes in the presence of the mirror and confirm the bonding hybridization of the mirror and the NP-dimer by mapping 3D surface charge distributions directly. Their near-field enhancement and resonance shifts have also been summarized. The system investigated here can provide a general idea and indication of the plasmon hybridizations and related near-field enhancement of metallic NPs on a metal mirror structures.

## Computational Method

**Figure 1:**
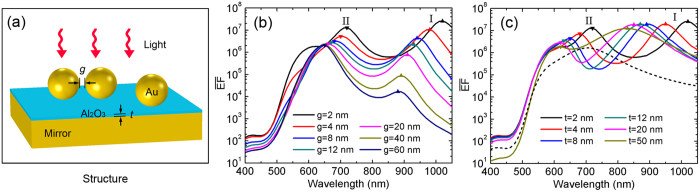
Schematic structure and calculated average near-field enhancement spectra. (**a**) Schematic structure of considered NPOM system. (**b**,**c**) FEM calculated near-field 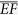 spectra by: (**b**) keeping *t* = 2 nm unchanged and varying *g*; (**c**) keeping *g* = 2 nm unchanged but varying *t* instead. The dashed black curve is the calculated spectrum for an isolated Au NP dimer with *g* = 2 nm.

3D electrodynamic calculations are performed using frequency-domain finite element method (FEM) in COMSOL Multiphysics software package (installed on a Quad Intel Xeon CPU, 64 GB RAM workstation). Typically, the studied structure consists of Au NPs (radius *R* = 60 nm) located randomly above a metal mirror, separated by a thin Al_2_O_3_ dielectric spacer, as is schematically depicted in Fig. 1(a). The mirror is set to be 100 nm in thickness, which is optically thick for metals in the visible regime. The Al_2_O_3_ layer can be prepared by atomic layer deposition (ALD) of oxides directly onto the mirror^27^,^54^. For simplicity, the NPDOM structure is considered first. The presence of a third NP won’t significantly change the NP-NP coupling and the near-field enhancement distribution of the system as the couplings between NPs relies on their separations^24^,^44^,^52^. In the process of simulation, the illumination is incident from the particle side, normal to the mirror, with a polarization along the dimer axis. The refractive index of Al_2_O_3_ is 1.62 while the metal dielectric functions ε(w) are modeled by a Lorentz-Drude dispersion model fitting the experimental data in Palik’s book^55^:


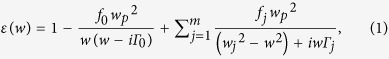


where *w*_*p*_ is the plasma frequency with oscillator strength *f*_*0*_ and damping constant *Γ*_*0*_. The last term of Eq. 1 is the result of the Lorentz modification, where *m* is the number of oscillators with frequency *w*_*j*_, strength *f*_*j*_ and damping constant *Γ*_*j*_. The fitting parameter values for gold are *f*_*0*_ = 0.760, *w*_*p*_ = 9.03 eV, *Γ*_*0*_ = 0.053 eV, *f*_*1*_ = 0.024, *Γ*_*1*_ = 0.241 eV, *w*_*1*_ = 0.415 eV, *f*_*2*_ = 0.010, *Γ*_*2*_ = 0.345 eV, *w*_*2*_ = 0.830 eV, *f*_*3*_ = 0.071, *Γ*_*3*_ = 0.870 eV, *w*_*3*_ = 2.969 eV, *f*_*4*_ = 0.601, *Γ*_*4*_ = 2.294 eV, *w*_*4*_ = 4.304 eV, *f*_*5*_ = 4.384, *Γ*_*5*_ = 2.214 eV, *w*_*5*_ = 13.32 eV^56^.

Meanwhile in practical applications, it has recently been fully appreciated that there exists a distinct deviation of spectral positions between the near- and far-field plasmonic responses as the near-field resonance is usually red-shifted compared to the far-field resonance, and in many cases only single or several fixed laser wavelengths are considered for the near-field enhancement^18^,^57^,^58^. To collect the entire near-field spectral characteristics and to extract the resonance wavelength where a maximum near-field enhancement is achieved, an average near-field enhancement spectroscopy is used in this paper^59^,^60^,^61^. The spectroscopy is obtained by averaging the volume integral of |***E***|^4^/|***E***_0_|^4^:


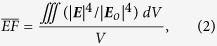


where *V* is the volume within a certain distance above the metal NP surface (here we take 2 nm)^61^, |***E***_**0**_| = 1 V/m is the modulus of incident field and ***E*** = (*E*_*x*_*, E*_*y*_*, E*_*z*_) is the local electric field. It is known that the enhancement factor (EF) of SERS is approximately proportional to the forth power of the local electric field intensity (|***E***|^4^/|***E***_0_|^4^)^62^,^63^. Thus the physical significance of 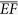 can be understood as the averaged EM EF of surface enhanced Raman scattering (SERS) on the assumption that adsorbed Raman probe molecules distribute randomly and uniformly at the surface of metal NPs. The far-field properties in terms of the extinction spectra are calculated for comparison (see Supplementary Fig. S2). As is known, 3D FEM is very computational expensive^64^. The computational time for an entire spectrum in this paper, e.g., 60 spectral points in the wavelength range of 400–1000 nm with 10 nm wavelength spacing is around 48 h. The highest spatial resolution of the grid is 0.5 nm at the gaps in all our simulations, resulting into more than three layers of grid within the gaps which can ensure the numerical accuracy to some extent, yet the calculated maximum electric field may be stronger using a finer meshing grid^24^,^65^.

To confirm the plasmon mode, 3D surface charge distributions are calculated by considering the skin effect and applying Gauss’ law during FEM calculations. Based on the skin effect, we assume that the induced charge density *ρ*_*r*_ is the largest at the metal surface *S* and decreases exponentially when spreading into the metal:


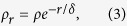


where *ρ* is the charge density at the surface, *r* is the depth from the surface and *δ* is the skin depth^66^,^67^,^68^. The total polarization charge *Q* = 0 within the metal NP is thus:


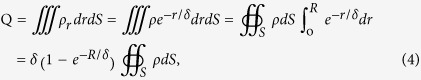


where *R* is the radius of the nanoparticle.

On the other hand, the Gauss’ law in the integral form is:


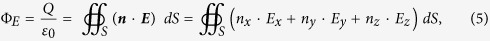


where 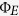 is the electric flux through the metal surface *S*, *ε*_*0*_ is the permittivity of vacuum,***n*** = (*n*_*x*_*, n*_*y*_*, n*_*z*_) is the outward normal vector of the metal surface and ***E*** = (*E*_*x*_, *E*_*y*_, *E*_*z*_) is the local electric field. The surface charge density can then be deduced by:


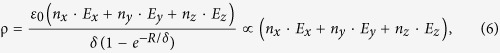


In the process of FEM calculations and plasmon mapping, (*n*_*x*_ ∙ *E*_*x*_ + *n*_*y*_ ∙ *E*_*y*_ + *n*_*z*_ ∙ *E*_*z*_) is used to indicate the surface charge density *ρ*. The use of this mapping approach makes it possible for us to acquire directly 3D surface charge distributions, which is ideally suited to recognize the geometry (or order) of complicated and hybridized plasmon modes^69^.

## Results and Discussion

### Average near-field enhancement spectra

**Figure 2:**
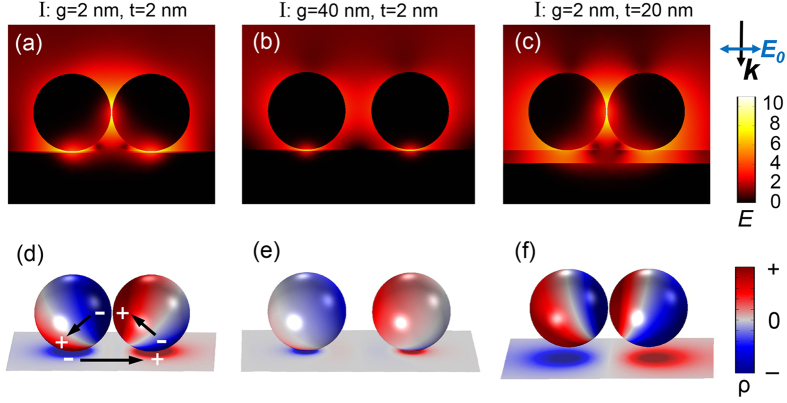
Plasmon mapping of MBDP mode I. (**a**–**c**) Typical local electric field distributions in the form of logarithmic |***E/E***_**0**_|^4^ for MBDP mode I. From left to right, (**a**) *g* = 2 nm, *t* = 2 nm, *λ* = 1020 nm; (**b**) *g* = 40 nm, *t* = 2 nm, *λ* = 890 nm; (**c**) *g* = 2 nm, *t* = 20 nm, *λ* = 850 nm. (**d**–**f**) 3D surface charge distributions corresponding to (**a**–**c**), respectively. Red color represents positive charge while blue is negative. Each black arrow indicates a dipole.

**Figure 3:**
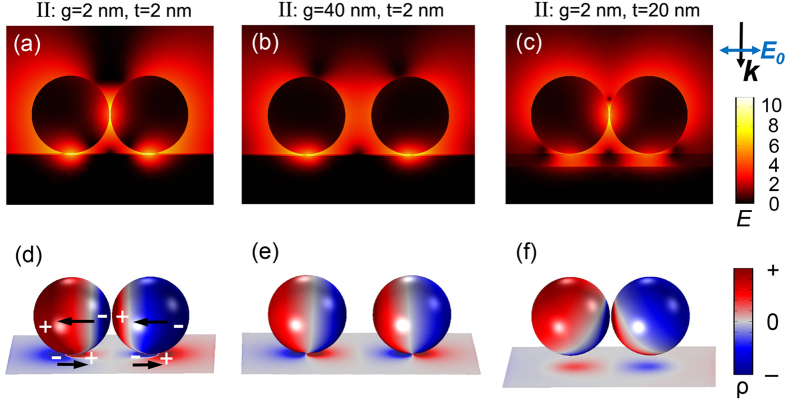
Plasmon mapping of MBDP mode II. (**a**–**c**) Local electric field distributions and (**d**–**f**) 3D surface charge distributions for MBDP mode II at shorter wavelengths *λ* = 720, 650 and 620 nm, respectively. The configurations and mapping method are the same as in Fig. 2.

The coexistence of near-field NP-NP and NP-mirror couplings present in the NPDOM model is investigated by varying the dimer gap *g* and the spacer thickness *t* as they significantly affect the strength of NP-NP and NP-mirror couplings, respectivley^18^,^27^. Figure 1(b) shows the calculated near-field 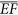 spectra by increasing *g* from 2 to 60 nm and keeping *t* = 2 nm unchanged, while in Fig. 1(c), *g* = 2 nm, *t* varies from 2 to 50 nm. It is noticed that there are two pronounced resonance peaks for each structure. In particular, the peak positions are *λ* = 1020 and 720 nm for NPDOM *g* = 2 nm, *t* = 2 nm. In both plots, those peaks indicated by up triangle symbols are indentified to be the same kind of plasmon mode (I) and the ones indicated by down triangle symbols belongs to another plasmon mode (II), which will be demonstrated in Figs 2 and 3. As either *g* or *t* increases, both modes exhibit a gradual blueshift and the near-field enhancement sustained by mode II undergoes a modest decrease. For mode I, the peak near-field enhancement decreases rapidly as *g* increases but stays nearly steady when *t* changes. These similarities and differences in response to changes of *g* and *t* factors are further analyzed in the following parts.

As a blank control, the NPDOM structures with mirror removed, i.e., isolated dimers have also been studied. To be specific, the dashed black curve in Fig. 1(c) is the calculated 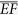 spectrum for an isolated dimer with *g* = 2 nm. The peak intensity 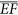 = 1.7 × 10^6^ occurs at *λ* = 705 nm, where the plasmon mode is confirmed to be the BDP mode (Fig. S1)^18^. Compared this spectrum with the solid black one (NPDOM *g* = 2 nm, *t* = 2 nm), we find surprisingly that not only is the mirror-induced peak 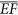 intensity (2.5 × 10^7^) much stronger than that of the isolated dimer, but also, over a broad wavelength range, the near-field enhancement of the NPDOM configuration is stronger than that of the corresponding isolated dimer. In other words, the near-field enhancement of a dimer can be further enhanced by adding a mirror and the NPDOM configuration offers an approach to raise the upper limit of the near-field enhancement based on conventional dimer systems. This additional enhancement mechanism can significantly benefit applications like single molecule detection^28^,^43^. To understand the extreme enhancement, the plasmon hybridizations are investigated.

### Plasmon mapping

For mode I, typical local electric field distributions at the resonance energies are shown in the upper panels of Fig. 2, in the form of logarithmic |***E/E***_**0**_|^4^ (i.e., EF). Figure 2(a–c) correspond to NPDOM configurations: (a) *g* = 2 nm, *t* = 2 nm; (b) *g* = 40 nm, *t* = 2 nm; (c) *g* = 2 nm, *t* = 20 nm from left to right, where the maximum EF = 1.3 × 10^11^, 1.0 × 10^9^, 1.3 × 10^10^ and 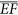 = 2.5 × 10^7^, 8.8 × 10^4^, 1.8 × 10^7^ respectively. It is easy to see that there are two kinds of hot-spots in Fig. 2(a): one is located at the NP-NP gap and the other is located at the NP-mirror gap. As *g* increases, both hot-spots degenerate rapidly (Fig. 2(b)), especially the NP-NP hot-spot. However when *t* increases, the NP-mirror hot-spot degenerates while the NP-NP hot-spot remains. Experimentally, the two kinds of hot spots can be precisely located using SERS spectroscopy by rationally choosing the probe molecules and excitation wavelengths^24^,^52^,^53^.

In the bottom panels of Fig. 2, the corresponding 3D surface charge distributions are plotted. It is worth mentioning that the plotted surface charge distributions are with the maximum transient charge polarizations within one full oscillation (See Supplementary Movies S1 and S2). We can observe a strong correlation between the surface plasmon geometry and the local electric field distributions. The mapping here shows clearly the fundamental dipole mode for individual NPs, the hybridized BDP mode in terms of the NP dimer, and a single dipole in the mirror, which are illustrated by black arrows in Fig. 2(d). Seen from the geometry of surface charge poles, we can confirm the plasmon mode as the same one, which is named mirror-induced BDP (MBDP) mode I for convenience. The image poles in the mirror are induced by neighboring opposite poles of the NPs on the mirror. Therefore, as *g* increases, the strongth of the NP-NP coupling soon becomes weaker, resulting into a weakened NP-mirror coupling and thus the degeneration of both hot-spots (Fig. 2(b)). Keeping *g* unchanged, the NP-mirror coupling can be further weakened by increasing the factor *t* (Fig. 2(c)).

Figure 3 shows the local electric field distributions and 3D surface charge distributions for MBDP mode II corresponding to each of the structures in Fig. 2. Still, both the NP-NP and NP-mirror hot-spots are observed in NPDOM *g* = 2 nm, *t* = 2 nm. The maximum EF = 2.5 × 10^11^, 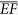 = 1.3 × 10^7^. The difference is that: as *g* increases, the NP-mirror hot-spot remains while the NP-NP hot-spot degenerates rapidly (Fig. 3(b)); when *t* increases, it is the NP-NP hot-spot that remains and the NP-mirror hot-spot degenerates (Fig. 3(c)). The plasmon modes are confirmed by the mapping in Fig. 3(d–f). Viewed from the NP dimers, the mode also belongs to the BDP mode, yet the orientations of individual dipoles differ from those in Fig. 2(d–f). A gradual plasmon evolution of mode II as *t* increases from 2 to 20 nm (keeping *g* = 2 nm) is shown in Fig. S3. There are two dipoles induced in the mirror as indicated by the black arrows in Fig. 3(d). Each NP on the mirror couples with its image dipole independently. Considering the total number of poles, MBDP mode II contains four dipoles (or a pair of NPOM quadrupole, see Fig. S4(b)) while mode I contains three diploes. Therefore mode II can be treated as a higher order plasmon mode with respect to mode I, and it also makes sense that mode II occurs at shorter wavelengths (higher energies)^70^.

As a conclusion, the extreme near-field enhancement induced by the mirror can now be qualitatively attributed to the bonding hybridizations of the propagating surface plasmon modes on the mirror surface and the localized surface plasmon mode of the NP-dimer, which is also found in the structure for SHINERS^52^,^70^. The NP-dimer offers one component: the bonding dipole plasmon (BDP) mode, while the mirror offers a dipole mode and a two-dipole mode (one order higher, occurs at shorter wavelengths), see the black arrows in Figs 2(d) and 3(d). Note that for the NPDOM configuration *g* = 2 nm, *t* = 2 nm, mode I and II take place at *λ* = 1020 and 720 nm respectively (Fig. 1), which are red shifted compared with the BDP mode of the isolated dimer (*λ* = 705 nm). This is consistent with the result predicted by the plasmon hybridization model^71^, i.e., a bonding mode occurs at longer wavelengths (or lower energies). Actually, seen from the orientations of their hybridized surface charge poles and the opposite poles in adjacent regions (Figs 2 and 3), the bonding property of the plasmon hybridizations can be concluded directly^61^.

### Peak near-field enhancement

**Figure 4:**
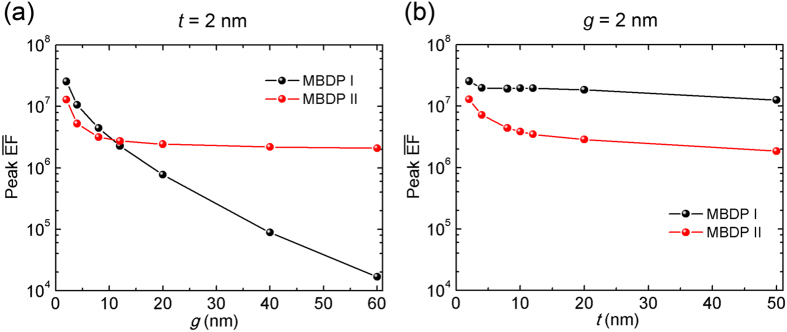
Peak near-field enhancement. (**a**,**b**) Extracted peak 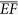 intensity of MBDP modes I and II as a function of: (**a**) the dimer gap *g*; (**b**) the spacer thickness *t*, respectively.

For further understanding on mirror-induced near-field enhancement and the plasmon resonances, the peak 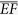 intensities of MBDP modes I and II in Fig. 1(b,c) are extracted and plotted in Fig. 4, as a function of the dimer gap *g* and the spacer thickness *t*, respectively. It is clear that for mode I, the responses on near-field enhancement to *g* and *t* changes are quite different. As *g* continues to increase, the peak 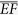 decreases almost exponentially, owing to a rapid degeneration of both NP-NP and NP-mirror hot-spots. But when *t* increases, it stays nearly steady at a relatively high intensity (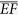 > 1.2 × 10^7^). Thus the total near-field enhancement of MBDP mode I is mainly determined by the NP-NP hot-spot while being affected little by the changes in the spacer thickness. Interestingly, noticing the blueshift of the resonance (Fig. 1(c)), it can be concluded that the change of *t* factor offers a strategy to easily tune mode I to a desired resonance wavelength in practical applications without decreasing its maximum near-field enhancement.

On the other hand, for mode II, it is found that both *g* and *t* decrease the peak 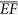 intensity slowly, approaching a stable value respectively. The total near-field enhancement is a result of synergistic effects of NP-NP and NP-mirror couplings^52^. Considering the evolutions of the NPDOM structure, as *g* increases endlessly, it is actually an isolated NPOM structure (See Fig. S4). And when *t* approaches infinity, it can be treated as an isolated NP dimer. In the latter case, the peak 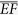 = 1.8 × 10^6^ for NPDOM *g* = 2 nm, *t* = 50 nm, which is indeed identical with the maximun intensity 1.6 × 10^6^ for an isolated NP dimer *g* = 2 nm. The detailed near- and far- field plasmonic properties for the isolated dimer system can be found in our previous work^18^. As for the former, the peak 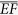 = 2.1 × 10^6^ for NPDOM *g* = 60 nm, *t* = 2 nm, coinciding with the peak 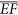 = 2.1 × 10^6^ for NPOM *t* = 2 nm which is shown by the black curve in Fig. S4(a).

### Near–field resonance shift

**Figure 5:**
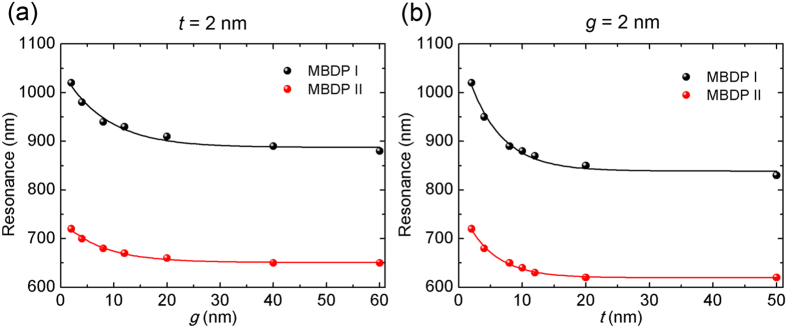
Near-field resonance shift. Extracted resonance wavelengths for both modes as a function of: (**a**) the dimer gap *g*; (**b**) the spacer thickness *t*, respectively. The smooth curves in both plots represent least-squares fits to the single-exponential decay function.

Considering the spectral deviation between the near- and far-field plasmonic responses^18^,^57^,^58^, it is of significant importance to clarify the near-field resonance shift in order to maximize the near-field enhancement at a specific excitation wavelength in practical applications. In Fig. 5, we summarize the near-field resonance wavelengths of MBDP modes I and II. Surprisingly, both modes are blue shifted with a lower speed as not only the dimer gap *g* but also the spacer thickness *t* increases monotonically, suggesting the consistency between NP-NP and NP-mirror couplings in terms of the nature of near-field coupling. The smooth curves in the plots are least-squares fits to single-exponential decay function:


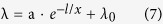


where *λ* is the resonance wavelength, *x* represents one of the two factors: *g* and *t*. The fitting parameters *a*, *l* and *λ*_*0*_ are listed in Table 1. For each configuration, the decay length *l* is within 10 nanometers. We also find that the resonance shift of MBDP mode I is of a relatively high decay length compared to that of MBDP mode II. The fitting here is encouraged by the plasmon ruler equation summarized for the far-field resonance shift^72^,^73^. It turns out to be suitable for the near-field coupling as well. Also a power law fit may be appropriate here^74^. Based on a quasistatic dipole coupling model, an intuitive picture of the distance decay of the near-field coupling in metal nanostructures can be presented^72^. Basically the dipole near-field of a plasmonic particle decays as the cube of the inverse distance. As a result, the near-field coupling strength in a dimer or NPOM system becomes a function of *g*^−3^ or *t*^−3^, a dependence which can be approximated very nearly to an exponential decay.

## Conclusion

In conclusion, we have performed a numerical study on the plasmon hybridizations and related near-field enhancement of metallic nanoparticle-dimer on a mirror structures. The coexistence of NP-NP and NP-mirror couplings is considered at normal incidence. By mapping their 3D surface charge distributions directly, we have demonstrated two different kinds of MBDP modes and confirmed the bonding hybridizations of the mirror and the NP-dimer which may offer a much stronger near-field enhancement than that of the isolated NP dimers over a broad wavelength range. It is further revealed that the near-field enhancement of these two modes exhibit different dependence on the NP-NP and NP-mirror hot spots. The total near-field enhancement of MBDP mode I that occurs at longer wavelengths compared to mode II is mainly determined by NP-NP hot-spots. For MBDP mode II, the total near-field enhancement is a result of synergistic effects of NP-NP and NP-mirror couplings instead. The near-field resonance wavelengths of both modes can be tuned to the blue exponentially by increasing the NP-NP gap or the NP-mirror separation. Our results here benifit significantly the fundamental understanding and practical applications of metallic NPs on a mirror structures in plasmonics.

## Additional Information

**How to cite this article**: Huang, Y. *et al*. Hybridized plasmon modes and near-field enhancement of metallic nanoparticle-dimer on a mirror. *Sci. Rep.* **6**, 30011; doi: 10.1038/srep30011 (2016).

## Supplementary Material

Supplementary Information

Supplementary Movie S1

Supplementary Movie S2

## Figures and Tables

**Table 1:** Fitting parameters for the exponential decay curves 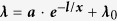 in Fig. 5(a,b).

Configuration	*a*	*l* (nm)	*λ*_*0*_ (nm)	*R*^2^
MBDP I vs. *g*	161.3	8.207	887.3	0.969
MBDP II vs. *g*	87.43	7.607	650.8	0.991
MBDP I vs. *t*	259.8	5.161	838.6	0.984
MBDP II vs. *t*	150.6	4.740	619.5	0.993
