# Supplementary material for: Hybridized plasmon modes and near-field enhancement of metallic nanoparticle-dimer on a mirror
Source: Sci Rep. 2016 Jul 15;6:30011. doi: 10.1038/srep30011 (PMC4945943; doi:10.1038/srep30011)
Supplement: Supplementary Information [file srep30011-s1.doc]

Supplementary Information for:

**Hybridized plasmon modes and near-field enhancement of metallic nanoparticle-dimer on a mirror**

Yu Huang,^1^ Lingwei Ma,^1^ Mengjing Hou,^1^ Jianghao Li,^1^ Zheng Xie^2^ & Zhengjun Zhang^3*^

^1^State Key Laboratory of New Ceramics and Fine Processing, School of Materials Science and Engineering, Tsinghua University, Beijing 100084, P. R. China,

^2^High-Tech Institute of Xi’an, Shaanxi 710025, P.R. China,

^3^Key Laboratory of Advanced Materials (MOE), School of Materials Science and Engineering, Tsinghua University, Beijing 100084, P. R. China.

Correspondence and requests for materials should be addressed to Z.J.Z. (email: zjzhang@tsinghua.edu.cn).

**Far-field Properties**. Extinction spectra are calculated by integrating the time-averaged extinction Poynting vectors ***S_ext_*** (i.e. electromagnetic power flow) over an auxiliary surface enclosing the Au NP dimer or the isoalted NP:


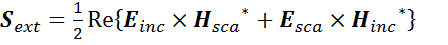
 (1)


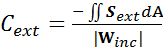
 (2)

where ***E***_inc_, ***E***_sca_, ***H***_inc_ and ***H***_sca_ are the incident and scattered electric and magnetic field respectively, ***C****_ext_* is the extinction cross section,
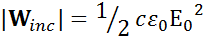
 is the power flow per unit area of the incident plane wave, *E_0_* (set at 1 V/m here) is the modulus of ***E***_inc_, *c* is the velocity of light and *ε_0_* is the permittivity of vacuum.


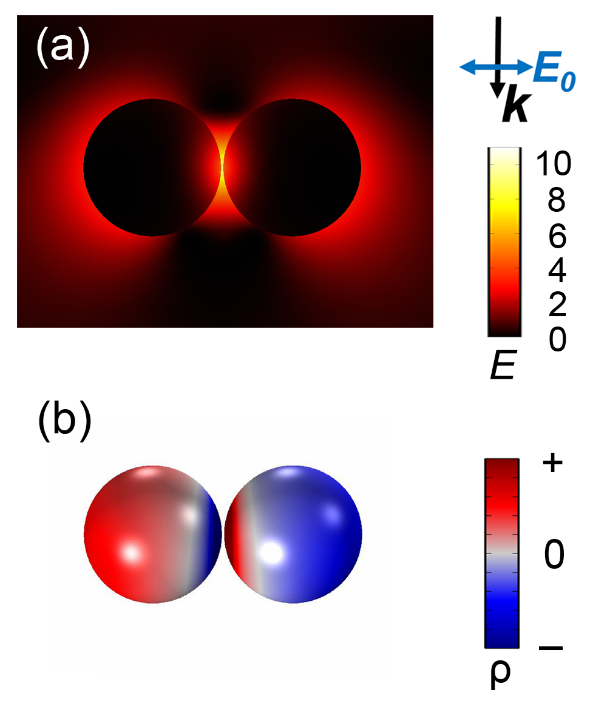


**Figure S1.** (a) Local electric field distributions in the form of logarithmic |***E/E*_0_**|^4^ for the isolated Au NP dimer: *g* = 2 nm, *λ* = 705 nm. (b) 3D surface charge distributions, indicating clearly the the BDP mode. This plasmon mapping corresponds to the peak of the dashed black curve in Fig. 1(c).


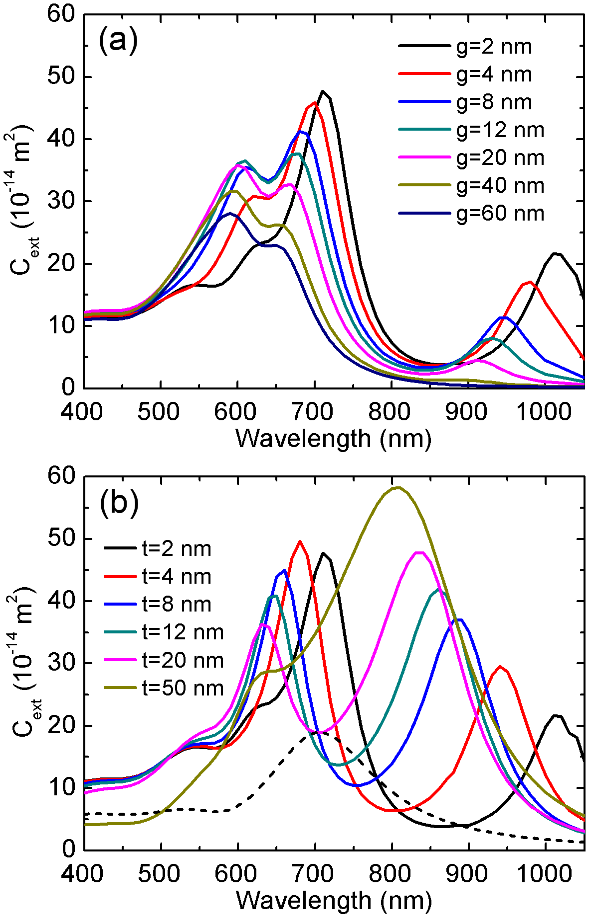


**Figure S2.** FEM calculated far-field extinction cross section *C_ext_* spectra for the same NPDOM configurations as discussed in Figs. 1(b) and 1(c), respectively. Regarding the peaks, there is a one-to-one correspondence between the far-field extinction and near-field
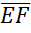
 spectra.


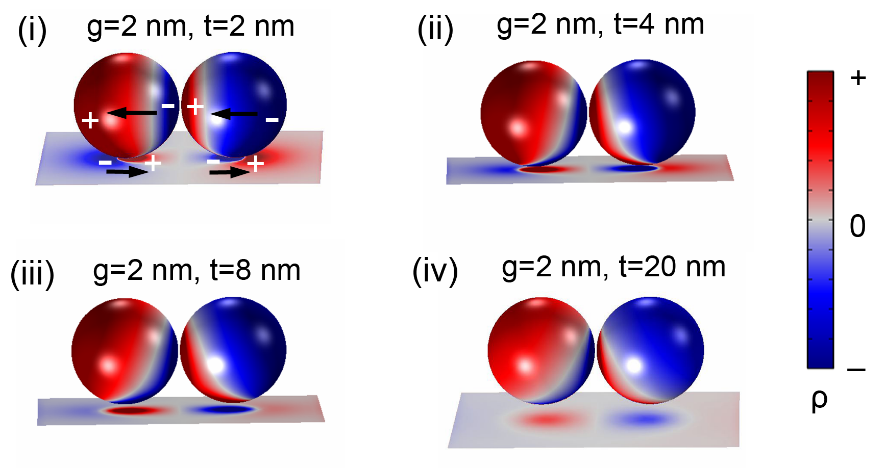


**Figure S3.** The gradual plasmon evolution of mode II as *t* increases from 2 to 20 nm (keeping *g* = 2 nm). (i) and (iv) correspond to Figs. 3(d) and 3(f), respectively. For *t* = 20 nm, the induced surface charge poles are very weak but there are still two pairs of negative and positive poles in the mirror. Thus the mode remains to be mode II.

**Isolated NPOM configurations.** In order to address the near-field coupling mechanism of MBDP mode II when the dimer gap *g* is very large and the NP-NP coupling becomes negligible[^3^](#_ENREF_3), the near-field enhancement of isolated NPOM configurations is considered. Fig. S4(a) shows the calculated near-field
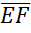
 spectra for the isolated Au NP (radius *R* = 60 nm) on Au mirror by varying the Al_2_O_3_ spacer thickness *t*. The resonance peaks are marked by down triangle symbols. As *t* increases, the resonance is first shifted to the blue gradually, but then back to the red a little. Meanwhile the peak
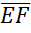
 decreases rapidly as is shown in Fig. S4(b). Typical local electric field distributions are plotted in Figs. S4(c) and S4(d). We can see that the maximum EF of NP-mirror hot-spot decays by nearly 5 orders of magnitude (from 1.6 × 10^10^ to 2.0 × 10^5^) as *t* increases from 2 to 20 nm. The inset in Fig. S4(b) is the 3D surface charge distributions for NPOM *t* = 2 nm at *λ* = 650 nm. An opposite dipole is generated in the mirror corresponding to the NP dipole above the mirror, which is in good accordance with the mapping for MBDP mode II in Fig. 3. Besides, the
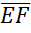
 intensity of the isolate Au NP (the black dashed curve in Fig. S4(a)) is much lower than that of mirror-coupled ones, i.e., NPOM configurations. These results demonstrate again that the coupling between the NP dipole and its image dipole is the key for the near-field enhancement of MBDP mode II. And as expected, it is rather sensitive to the dielectric spacer thickness[^4^](#_ENREF_4).


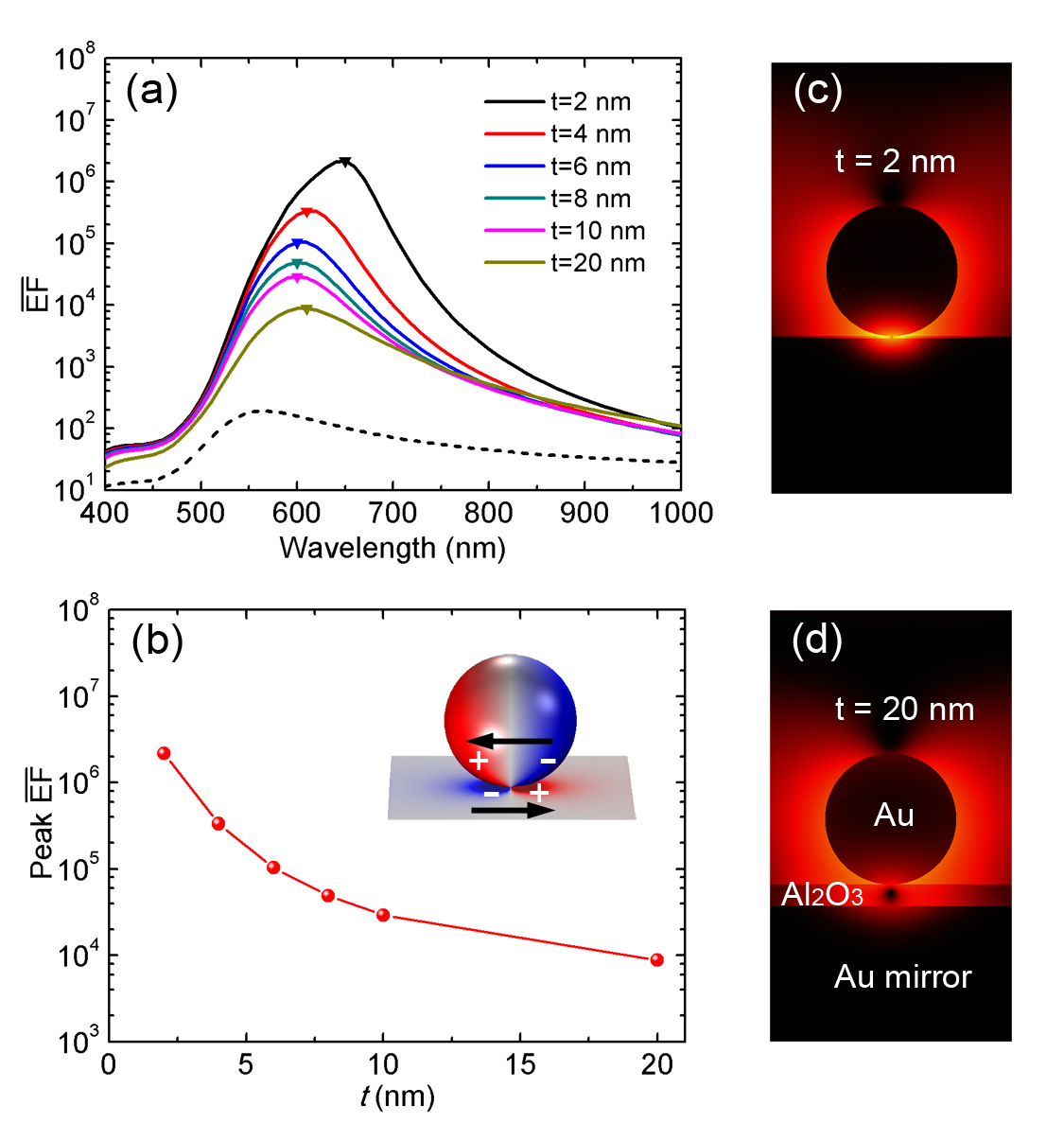


**Figure S4 | Near-field enhancement and plasmon mapping for isolated Au NPOM structures.** (a) Calculated near-field
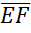
 spectra for isolated Au NPOM structures. The black dashed curve is the calculated spectrum for the isolate Au NP. (b) Extracted peak
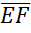
 intensity as a function of *t*. The inset is the mapping of 3D surface charge distributions for NPOM *t* = 2 nm at *λ* = 650 nm. (c)-(d) Typical local electric field distributions for: (c) *t* = 2 nm, *λ* = 650 nm; (d) *t* = 20 nm, *λ* = 610 nm. The E-field legend is the same as in Figs. 2 and 3.

**Supplementary Movies | 3D surface charge distributions within on oscillation as the phase of incident field varies from 0 to 2π**.

**Movie S1** corresponds to the mapping in Fig. 2(d): MBDP mode I, *λ* = 1020 nm, *g* = 2 nm, *t* = 2 nm. The legend shows the value of the surface charge density *ρ*, red color represents positive charge while blue is negative. The conduction electrons are driven by the oscillating electric field of light.

**Movie S2** is for Fig. 3(d): MBDP mode II, *λ* = 720 nm, *g* = 2 nm, *t* = 2 nm.

**References**

1 Giannini, V., Fernandez-Dominguez, A. I., Heck, S. C. & Maier, S. A. Plasmonic Nanoantennas: Fundamentals and Their Use in Controlling the Radiative Properties of Nanoemitters. *Chem. Rev.* **111**, 3888-3912 (2011).

2 Bohren, C. F. & Gilra, D. P. *Absorption and Scattering of Light by Small Particles*. (Wiley, 1983).

3 Huang, Y. *et al.* Nanogap effects on near- and far-field plasmonic behaviors of metallic nanoparticle dimers. *Phys. Chem. Chem. Phys.* **17**, 29293-29298 (2015).

4 Mubeen, S. *et al.* Plasmonic Properties of Gold Nanoparticles Separated from a Gold Mirror by an Ultrathin Oxide. *Nano Lett.* **12**, 2088-2094 (2012).
